# PROTOCOL: Prevention, Intervention, and Compensation Programs to Tackle School Dropout: An Evidence and Gap Map

**DOI:** 10.1002/cl2.70032

**Published:** 2025-03-13

**Authors:** Marta Pellegrini, Carmen Pannone, Daniela Fadda, Laura Francesca Scalas, Giuliano Vivanet, Amanda Neitzel

**Affiliations:** ^1^ Department of Education, Psychology, Philosophy University of Cagliari Cagliari Italy; ^2^ School of Education, Center for Research and Reform in Education Johns Hopkins University Baltimore Maryland USA

**Keywords:** early school leaving, evidence and gap map, school dropout

## Abstract

The issue of students dropping out before completing secondary education is a global concern with significant individual and societal consequences. Various terms, such as Early School Leaving (ESL), Early Leaving from Education and Training (ELET), and school dropout, reflect different policy perspectives on this phenomenon. Despite international efforts to address school dropout, a comprehensive review identifying areas with strong evidence and research gaps is lacking. This Evidence and Gap Map (EGM) systematically reviews the evidence on programs aimed at reducing school dropout and improving intermediate outcomes (e.g., educational performance, attendance). Following the 2011 European Council Recommendations, interventions are categorized into three domains: prevention, intervention, and compensation. The EGM will provide school and policy decision‐makers, as well as program developers, with an overview of research evidence useful for guiding efforts to reduce school dropout and design effective programs. By pinpointing research gaps, the EGM will help identify critical areas where further investigation is needed to better understand and address the impact of school dropout programs.

## 1 Background

### 1.1 The Problem, Condition or Issue

The problem of students dropping out before completing secondary education is a matter of global concern. *Early School Leaving* (ESL), *Early Leaving from Education and Training* (ELET), and *school dropout* are the terms most used by international and national organizations to define this phenomenon. These terms and the related indicators reflect different perspectives and policy focuses. For instance, in 2003, the European Union adopted the term “Early School Leavers” to refer to individuals “aged 18 to 24 with only lower secondary education or less and not in education or training” (Council Conclusion 2003/C 134/02), only to later define them as “Early Leavers from Education and Training” (ELET), because this concept more clearly includes those who have dropped out of vocational training tracks (European Commission et al. 2019). Similarly, in the United States., the *school status dropout rate* is defined as “the percentage of 16‐ to 24‐year‐olds who are not enrolled in school and have not earned a high school credential” (National Center for Education Statistics [NCES] 2023). The NCES also tracks event dropout rates, status completion rates, and adjusted cohort graduation rates (i.e., students who graduate on time with a regular diploma). Several primary studies evaluate the impact of interventions at the end of secondary school by measuring dropout or graduation rates, as they are typically unable to track the attainment of qualifications years later (e.g. when participants reach the age of 24). Thus, we use more the definition by De Witte et al. (2013, 14) for the scope of our EGM: “school dropout has been defined as leaving education without obtaining a minimal credential (most often a higher secondary education diploma).”

International organizations and national policies consistently focus on establishing new targets and priorities to tackle dropout. The Incheon Declaration for Education 2030 – held by UNESCO in 2015 and signed by 160 countries – proposed Sustainable Development Goals to be achieved by 2030. Among them, Goal 4 aims to “ensure inclusive and equitable quality education and promote lifelong learning opportunities for all” (UNESCO 2016, 29). This target is monitored in each participating country through multiple indicators, including school‐level completion rate and out‐of‐school rate. As another example, the 2021 European Council Resolution set the target of reducing the school dropout rate to below 9% by 2030 (Council Resolution 2021/C 66/01). According to the latest estimate from UNESCO, approximately 244 million children and youths between the ages of 6 and 18 were out of school in 2021, with different distributions according to age group and world region. The highest percentage of dropouts was in upper secondary school, with 30% of youths aged 15–18 out of school. At lower secondary and primary school levels, 14% and 9% of children were out of school, respectively. Europe and North America had the lowest rates for each age group, while Sub‐Saharan Africa had the highest rates (Global Education Monitoring Report Team and UNESCO Institute for Statistics 2022). In Europe, the average rate of ELET in 2022 was 9.6%, which is 0.6 percentage points higher than the 2030 target (Eurostat 2023). Despite an overall declining trend in Europe (European Commission 2023), progress has not been equally distributed. The proportion of early school leavers ranged from 2.3% in Croatia to 15.6% in Romania, with high variations within and between EU Member States. These variations highlighted significant disparities among Europe, with 18 countries already meeting the 2030 target and nine countries experiencing an increase in the percentage of early school leavers (Eurostat 2023).

Dropping out of school before achieving a qualification has wide‐ranging implications for individuals and society. At an individual level, school dropout is associated with elevated rates of substance abuse, antisocial behaviors, criminal activity, and restricted career prospects (Audit Commission 2010; OECD 2023). It is also correlated with higher rates of poor mental and physical health (European Education and Culture Executive Agency, Eurydice 2014). At a societal level, individuals who drop out of school are less likely to engage in democratic processes and more likely to experience marginalization and unemployment (Dale 2010; OECD 2023), which have enormous economic implications. In the long run, consequences at a national level include increased welfare expenditures in healthcare and social services (Audit Commission 2010; International Labour Organization 2019; OECD 2023).

Initiatives at global, regional, and national levels have been implemented to address school dropout. For instance, “Safe Back to School,” created by Save the Children in 2020, aimed to support the return of over 150 million of the most marginalized children safely back into learning after the COVID‐19 pandemic (Save the Children 2023). As another example, the Nordic Council of Ministers, involving Norway, Sweden, Iceland, Finland, and Denmark, commissioned the *Nordic Web Resource on Dropout from Upper Secondary Education* project. Its goal is to collect effective initiatives to increase the rate of upper high school completion (Tägtström and Olsen 2016). Structured interventions have also been developed with the aim of reducing school dropouts, especially in Anglo‐Saxon countries. For example, the National Dropout Prevention Center in the United States offers a database of programs designed to address school dropout. Additionally, the What Works Clearinghouse regularly updates reviews of studies focusing on the effects of educational programs to address dropout, rating the magnitude of their impact and the strength of their evidence.

Evaluating the effectiveness of school dropout programs is challenging, as it demands longitudinal assessments to gauge their long‐term impact on dropout rates. If a program is implemented in elementary or lower secondary school, it is unlikely that a study will track students until the end of secondary school, due to the high costs and challenges involved in following students for a long time. Consequently, many studies focus on precursors or predictors of dropout, such as student academic achievement, attitudes, and behaviors. Rumberger (2011) provides a conceptual framework aimed at understanding the path that leads to school dropout and identifies the most important individual, family, and school risk factors. A description of the logic model is provided in Section 3.4. Our Evidence and Gap Map (EGM) addresses these challenges by identifying areas where substantial evidence exists on the impact of school dropout prevention, intervention, and compensation programs on measures of long‐term outcomes (i.e., school dropout and completion/graduation), along with intermediate outcomes, which include risk factors or precursors of dropout (e.g., educational performance, behaviors).

### 1.2 The Intervention

This EGM focuses on all interventions aimed at reducing school dropout or supporting school completion/graduation. Based on the 2011 European Council Recommendations, updated in 2022, we will categorize the programs into three broad domains: prevention, intervention, and compensation programs (Council Recommendation 2022/C 469/01 2022).

Prevention programs aim to reduce the risk of school dropout before the problem starts, optimizing education opportunities offered to all students and removing any obstacles to school success throughout grade levels. These measures include the early identification of risk factors, and the use of learner‐centered and inclusive curricula, including academic, social, and emotional education. Examples of programs in this category are high‐quality instructional strategies, teacher professional development, family engagement programs, and schoolwide approaches. These programs address all students to promote attendance and a culture of the importance of going to school (Balfanz et al. 2007). Intervention programs aim to avoid students dropping out of school by reacting to early warning signs and providing specific support for students at risk. This category of programs may address all grade levels, from early childhood to secondary school. However, it includes support programs targeting only students identified as at risk of underachievement, facing learning challenges, or broader multifaceted difficulties. Examples are mentoring practices, behavioral interventions, and supplemental services. The intensity of these programs varies according to students' needs. Targeted programs, such as those utilizing data tracking, typically involve 15%–20% of the student population. For students who do not respond to targeted interventions, more intensive programs, such as one‐to‐one mentoring, are implemented and involve approximately 5%–10% of the students (Balfanz et al. 2007). Compensation programs aim to address and re‐engage individuals who have already dropped out of school without earning a secondary school qualification. These programs offer them alternative ways to reach a qualification. Examples of this category of programs include Vocational Education and Training and alternative schools, such as General Equivalency Diploma preparation programs, for students who have left regular schools.

Within the three domains, eligible interventions for our EGM are school‐based, school‐affiliated, and community‐based programs that perform actions with the expectation of having a beneficial impact on staying in or returning to school. School‐based programs are those that take place under the control of school authorities. School‐affiliated programs are those that take place before or after school hours with the support of external professionals. Community‐based programs are services that are implemented at a local level, outside of the schools (Wilson et al. 2011).

### 1.3 Why It Is Important to Develop the EGM

Developing this EGM is important for several reasons. First, EGMs are valuable resources for policymakers, program developers, and researchers to inform the design, implementation, and evaluation of effective programs. By using a visual and accessible tool to show the extent of the evidence base, EGMs can support the discoverability of evidence and its use to make informed decisions in practice and policy. Our EGM will provide a comprehensive and up‐to‐date overview of studies that evaluated the impact of dropout prevention, intervention, and compensation programs on reducing school dropout rates and improving intermediate outcomes (see Section 3.4). Second, no previous EGMs on interventions to tackle school dropout have been published. Several EGMs exploring the impact of interventions related to student well‐being (Saran et al. 2020a), violence (Pundir et al. 2020), and inclusion (Saran et al. 2020b) considered school dropout or school enrollment as outcomes. However, the primary focus was on interventions other than programs directly aimed at reducing school dropout. The most recent systematic reviews with meta‐analysis on the impact of school dropout interventions were published in the 2010s (Chappell et al. 2015; Freeman and Simonsen 2015; Tanner‐Smith and Wilson 2013; Wilson et al. 2011). Wilson et al. (2011) included 167 impact studies that evaluated the effect of general prevention and intervention programs, as well as those targeting teen parents, for increased school completion. Tanner‐Smith and Wilson (2013) examined the effect of dropout programs on student absenteeism across 74 randomized and quasi‐experimental studies. Chappell et al. (2015) evaluated the effect of 61 dropout prevention programs on dropout rates and graduation rates. Freeman and Simonsen (2015) address research questions similar to the ones of our EGM. The recent meta‐analysis by Wang et al. (2024) included 26 studies conducted between 2010 and 2022, using similar eligibility criteria to Wilson et al. (2011). Although this is a relevant up‐to‐date resource, the overall lack of recent reviews on the impact of school dropout programs shows the need to conduct a comprehensive exploration of the evidence available through an EGM. Thirdly, our EGM will have a broad scope, including dropout compensation programs, which have not been considered in previous syntheses.

## 2 Objectives

The primary objective of this EGM is to evaluate the nature, extent, and robustness of evidence on school dropout programs.

The four secondary objectives are as follows:

i. To systematically catalog impact evaluations and systematic reviews with meta‐analysis of programs aimed at reducing school dropout, increasing school completion, and enhancing intermediate outcomes identified as precursors of school dropout (e.g., academic achievement, behaviors).
ii. To create a map of effectiveness studies that shows the extent of the evidence base and research gaps by intervention and outcome domains. Filters will be used to refine the information based on the PICOS (Population, Intervention, Comparison, Outcome, Study design) framework. By pinpointing research gaps, we can identify critical areas where further investigation is needed to better understand and address the impact of school dropout programs.
iii. To identify clusters of evidence suitable for future meta‐analyses. This will involve grouping studies by intervention domain (i.e., prevention, intervention, compensation) and outcome (i.e., long‐term and intermediate outcomes) to further explore the magnitude of results in more homogenous groups.
iv. To provide school and policy decision‐makers, as well as program developers, with an overview of research evidence useful for guiding efforts to reduce school dropout and design effective programs.

## 3 Methods

### 3.1 EGM: Definition and Purpose

An EGM is defined as a “systematic evidence synthesis product which displays the available evidence relevant to a specific research question” and is “used to identify gaps requiring filling with new evidence, collections of studies for review and increase the discoverability and use of studies by decision‐makers, research commissioners and researchers” (White et al. 2020, 1). Based on the EGM results, decision‐makers and commissioners may be facilitated in making evidence‐informed policies, as well as commissioning relevant research, based on the identified gaps. Program developers may be oriented to develop new approaches that have the best chance of being effective, based on the available evidence. Researchers may direct their efforts to investigate areas that need further development. It is worth noting that, while EGMs offer a visual overview of the available evidence, providing information on the impact of the interventions studied is outside of their scope. Further analyses are needed to code the study results and synthesize the magnitude of the effects.

### 3.2 Framework Development and Scope

Creating the EGM framework is recognized as the initial and crucial step in developing an evidence map (White et al. 2020). We will follow the typical framework of a matrix with interventions/programs in rows and outcomes in columns to develop our EGM. After drafting the framework based on the previous literature, we will review it through consultation with stakeholders. The process is outlined as follows. First, we will draft the framework, with the program and outcome domains and categories based on previous policy and research literature. Programs will be grouped into three broad domains—prevention, intervention, and compensation—based on the framework outlined by European Council Recommendation 2022/C 469/01. Each domain will be further distinguished into program categories, which we will identify through consulting the practice guide on preventing school dropout by the Institute of Education Sciences (Rumberger et al. 2017), the database of programs by the National Dropout Prevention Center (https://dropoutprevention.org/), and the Campbell Collaboration review conducted by Wilson et al. (2011). These three resources include a wide range of programs aimed at tackling school dropout. The categories will be updated according to the programs evaluated in the studies included in our review and their characteristics. Outcomes will be grouped into two broad domains (i.e., long‐term outcomes and intermediate outcomes) to distinguish between studies that directly evaluated the effects on school completion or dropout—long‐term outcomes—and studies that evaluated the effects on precursors of school dropout—intermediate outcomes (Battin‐Pearson et al. 2000; Rumberger 2011). Intermediate outcomes will be further distinguished into categories based on Rumberger's (2011) conceptual framework of individual risk factors, including areas of educational performance, problem behaviors, attendance, attitudes, and relational and social factors. The categories will be updated according to the outcomes considered in the studies. Program and outcome domains and categories are drafted in Annexes 1 and 2.

Second, the draft framework will be revised through a consultative process involving the authors of the map, members of the advisory group, school stakeholders, and local administrators. A workshop with stakeholders and local administrators will be organized to review the framework, with the aim of making the EGM easy to use for practitioners and policymakers. We will also ask for adjustments to the labels used to categorize programs and outcomes and their definitions to ensure that clear and easily understandable language is used.

### 3.3 Stakeholder Engagement

We have engaged research and school experts who agreed to contribute to the project as members of the Advisory Group. The Advisory Group has national and international members with different areas of expertise. We will also engage with teachers, principals, and local administrators in a workshop to advise on the EGM framework.

Therese Deocampo Pigott is a Professor at Georgia State University and an expert in methods for evidence synthesis and meta‐analysis.

Daniel Princiotta is an Assistant Research Scientist at Johns Hopkins University and an expert in school dropout prevention and recovery policies, practices, and interventions.

Veronica Mobilio is the Director of the Research Unit at Fondazione per la Scuola (FpS), an Italian training and research organization strongly committed to improving the connection between research and practice by making better use of available evidence.

The members of the Advisory Group will advise and review the EGM protocol and the final paper, supporting the quality of the process, the development of the EGM framework, and the interpretation of the results. Once we have a draft of the map, we will engage with teachers, principals, and local administrators in a workshop to advise on the EGM framework and share suggestions on how to make the EGM usable for stakeholders.

### 3.4 Conceptual Framework

**Figure 1:**
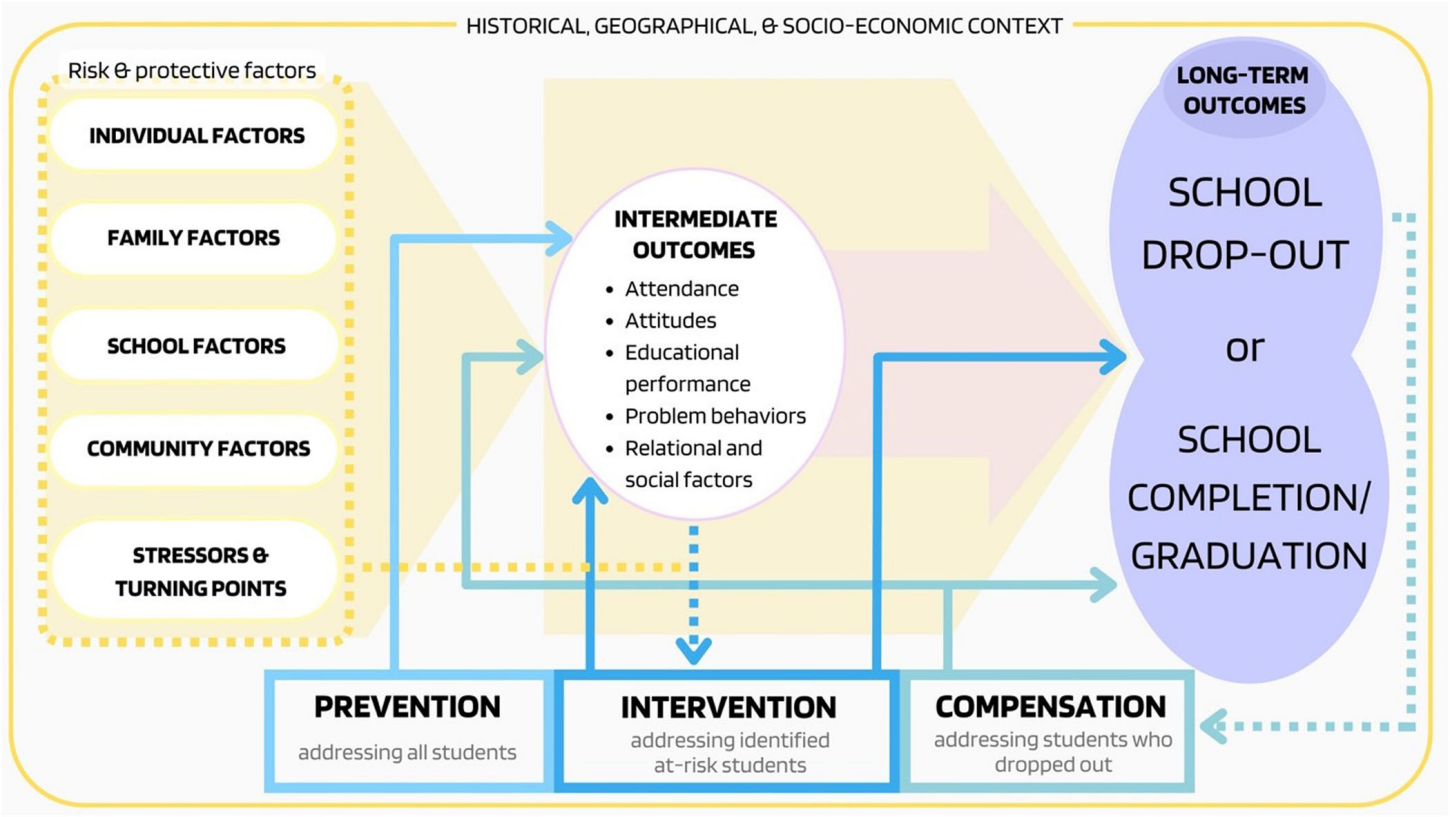
Logic model.

Research has shed light on how school dropout is often the long‐term manifestation of an individual and academic discomfort, evident much earlier through dropout risk factors (Battin‐Pearson et al. 2000; European Agency for Special Needs and Inclusive Education [EASNIE] 2019). We adopted a broad logic model to portray the general pathway expected for the impact of the programs on precursors of school dropout and, ultimately, on dropout rates. Figure 1 shows our logic model, which is based on the following: Rumberger's (2011) conceptual framework, which identifies the most important individual and institutional risk factors of school dropout; Dupéré et al.'s (2015) review and the EASNIE (2019) eco‐systemic approach to preventing school failure, which suggests a set of risks and protective factors at national, school, and individual levels; and Battin‐Pearson et al. (2000), who tested five models to predict school dropout and identify precursors, such as academic achievement and attendance.

In our logic model, programs designed to address school dropout are expected to have an impact on intermediate outcomes and long‐term outcomes. These programs operate through distinct pathways based on the three domains and their corresponding program categories, with direct effects on the long‐term outcomes or effects mediated by intermediate outcomes. The former outcome category is distinguished in the model as school dropout and school completion/graduation. The latter is organized according to the following target areas of student development: (a) educational performance, including academic achievement, educational persistence, and attainment (e.g., grade retention); (b) problem behaviors, including factors that reflect what students do in and outside of school (e.g., school suspension, school expulsion, deviant behaviors, substance use); (c) attendance, defined as a student being present in an accredited organized learning environment at any time during the school year; (d) attitudes, including a wide range of psychological factors, such as self‐efficacy, self‐esteem, learning motivation, and engagement; (e) relational and social factors, related to student–teacher interactions, peer relationships, and family relationships.

As mentioned above, we expect different pathways for the three program domains. Based on our logic model, prevention programs targeting all students have an impact on the intermediate outcomes, which, in turn, have a long‐term effect on the long‐term outcomes. This pathway finds support in the nature of this type of program, which aims to reduce the risk of school dropout before the problem manifests. Intervention programs target students identified as at risk of school dropout. The identification of such students typically involves using data from several variables, including individual, family, school, and community factors, along with intermediate outcomes, such as student attendance and educational performance. The dashed arrow connecting intermediate outcomes and other factors to intervention programs illustrates this pathway. Intervention programs have a direct impact on long‐term outcomes, as well as a mediated impact through intermediate outcomes. The intensity of intervention programs for the identified at‐risk students varies according to their individual needs. Targeted programs, typically delivered in small groups, reach a larger section of student populations, while intensive one‐to‐one programs involve a smaller section.

In contrast to prevention and intervention domains, compensation programs take a distinct trajectory, as they address individuals who have already dropped out of school. The objective of these programs is to re‐engage them by providing ways to earn a qualification. Similarly to intervention programs, we expect a direct impact of compensation programs on school completion/graduation and a mediated impact through intermediate outcomes. In Figure 1, this trajectory is depicted by an arrow connecting the long‐term outcome of school dropout to compensation programs, which, in turn, are linked to intermediate outcomes and school completion/graduation.

The characteristics of students who drop out of high school, along with their associated risk factors, have been extensively researched (e.g., Freeman and Simonsen 2015; Rumberger 2011). Although no individual risk factor accurately predicts dropout, the prediction becomes more accurate when multiple risk factors are considered. Thus, the presence of multiple risk factors significantly increases the likelihood that a student will leave school before achieving a secondary qualification (Freeman and Simonsen 2015). Our logic model considers these factors—either protective or risk factors—influencing the pathway to school dropout, including the above‐mentioned intermediate outcomes alongside individual and family factors, such as socioeconomic status (SES), gender, parents' level of education, and family structure, as well as school and community factors, such as school structure, school resources, urbanicity level, and employment opportunities (Rumberger 2011). We expect that a limited number of studies will provide information about these factors. Thus, we will code the ones that are commonly reported in studies to describe the population of interest (e.g., SES). We will use the filter function of the interactive EGM to show the results for different populations of students based on these factors.

The model also incorporates the contribution of Dupéré et al. (2015), which integrates the traditional life course framework with the stress process framework, emphasizing the interplay between long‐term risks and proximal life events and circumstances in the dropout pathway. School‐aged youths may face *stressors*— external events and circumstances that challenge their adaptive capacities through either disruptive isolated events or prolonged difficulties. Additionally, they may encounter *turning points*—significant departures from their past development brought on by events powerful enough to reshape their lives. Finally, the historical, geographical, and socioeconomic context frames the entire logical model. Lives are embedded in specific contexts that determine the conditions under which schools operate, and these contexts may influence the immediate environment in which the decision to drop out is made (Dupéré et al. 2015).

### 3.5 Dimensions

The EGM will be presented as an online matrix of intersections between programs (in rows) and outcomes (in columns). Programs and outcomes will be grouped into broad domains and categories. Each study will be allocated to the appropriate cell according to the evidence it presents, resulting in numerous appearances of the studies on the map. Bubbles, representing groups of studies of the same type, will have the following: (a) a different color based on the study design (systematic reviews with meta‐analysis, Randomized Controlled Trials [RCTs], Quasi‐Experimental Designs [QEDs]); and (b) a different size based on the number of studies. In addition to interventions and outcomes, several variables related to the PICOS framework will be coded and included in the EGM as filters to support the selection of interventions matching certain criteria of interest. We plan to include the population and study design characteristics described in the *Filters for presentation* section. EPPI Mapper will be used to generate an online interactive EGM.

#### 3.5.1 Types of Study Design

The following study designs are eligible for inclusion, as they are appropriate designs for supporting a causal inference on the effectiveness of educational interventions. We will include primary studies or evidence syntheses on group designs that compared the results of a treatment group implementing the intervention under evaluation with a control group using the regular practice or an alternative program. Single‐group designs (e.g., pretest‐posttest designs, interrupted time series) are not eligible for inclusion due to their lack of a comparison group. In such studies, the results may not be attributed solely to the treatment, as other factors, such as student maturation between the pretest and posttest, could also explain the observed differences.

a. *Systematic reviews with meta‐analysis*
  Reviews were conducted using transparent and reproducible methods to systematically synthesize quantitative studies on the impact of interventions addressing school dropout. To be eligible, the reviews must include experimental or quasi‐experimental studies and use meta‐analysis techniques to combine the study results.
b. *Experimental designs*
  RCTs in which individuals or clusters of individuals are randomly assigned to experimental and control conditions. Studies may include outcomes collected at various time points.
c. *Quasi‐experimental designs*
  QEDs comparing at least one treatment group with a comparison group, in which randomization to conditions was not possible (Shadish et al. 2002). We will include nonequivalent control group designs that matched treatment and comparison groups based on relevant characteristics, using simple matching or propensity score matching. Matching may happen prospectively or retrospectively (i.e., ex post facto or post hoc designs), namely before or after program implementation. In the case of post hoc designs, studies that matched treatment and comparison individuals using data from different years (e.g., data for treatment individuals in 2010 and data for comparison individuals in 2008) will be excluded. We will also include Difference‐in‐Difference (DiD) approaches, which compare outcomes over time between treatment and control conditions; Regression Discontinuity Designs (RDD), in which participants are assigned to conditions based on a cutoff or a threshold; and Instrumental Variable (IV) analyses, “a statistical technique for estimating causal relationships when an RCT is not feasible or when an intervention does not reach every participant/unit in an RCT” (White and Sabarwal 2014, i). For all QEDs, studies may include outcomes collected at various time points.

#### 3.5.2 Types of Intervention/Problem

The EGM will focus on programs where the goal is to tackle school dropout, categorizing them into prevention, intervention, or compensation program domains (see Annex 1 for a draft of program domains and categories). Within these domains, eligible interventions are school‐based, school‐affiliated, and community‐based programs that perform actions with the expectation of having a beneficial impact on staying in school (Wilson et al. 2011), as described in the *Intervention* section. Studies on educational policies or types of schools that cannot be adopted and implemented by any school (e.g., catholic vs. public schools) will be excluded. A large number of programs in the prevention and intervention categories are developed to address intermediate outcomes while considering reducing dropout rates as a long‐term goal. For example, kindergarten programs that primarily aim to enhance early reading skills, with additional support to at‐risk students, often indicate school dropout prevention as a long‐term goal. In this case, we will include these programs only if dropout/school completion rates are also measured in the studies. Other interventions may specifically focus on reducing school dropout rates, but the studies conducted could only measure intermediate outcomes. In this case, we will include studies reporting intermediate outcomes if it is explicit that the primary goal was to address school dropout.

#### 3.5.3 Types of Population

For prevention and intervention programs, the target population includes students who were attending K‐12 during the study. For compensation programs, the target population includes school leavers up to 24 years old. We considered the European Council's definition of early school leavers as a basis for setting the upper age limit at 24 (Council Recommendations 2011/C 191/01). Eligible dropout programs may target students, their teachers (e.g., teacher professional development programs), parents, school leaders, and other relevant stakeholders, but must evaluate student outcomes. Studies exclusively targeting students with disabilities were excluded to maintain focus on interventions applicable to the general student population, as these students face distinct challenges that require specialized programs, such as individualized education plans or assistive technologies, which differ significantly from standard dropout prevention strategies. However, studies addressing students with disabilities and their peers in mainstream educational settings are included.

#### 3.5.4 Types of Outcome Measures

Eligible outcome variables include long‐term outcomes on school completion/graduation and dropout, as well as intermediate outcomes that are found to be precursors of school dropout according to the literature (Battin‐Pearson et al. 2000; Rumberger 2011). Intermediate outcomes will be grouped into categories based on individual risk factors, including areas of educational performance, problem behaviors, attendance, attitudes, and relational and social factors (see Annex 2 for a draft of outcome categories). Studies measuring intermediate outcomes will be included under one of the following two conditions: when the program does not explicitly address reducing dropout rates but also reports long‐term outcomes, or when the program explicitly targets dropout reduction but could only measure intermediate outcomes.

### 3.6 Other Eligibility Criteria

#### 3.6.1 Types of Settings

No geographical restrictions will be applied to the studies, but only manuscripts available in English are eligible. This decision is based on practical considerations: most of the research is published in English, including journals and conference proceedings; the research team is proficient in English but not in other languages. We recognize that this is a limitation of our study and will address it as such in the final paper.

We will search and include both published and unpublished studies from 2010 to 2024. The decision to limit the search to studies from 2010 onward is based on the Campbell Collaboration review by Wilson et al. (2011), which thoroughly synthesized the evidence available until that time. Our review builds on Wilson's work to update the findings with more recent studies, allowing readers to refer to Wilson for older literature. Additionally, this approach ensures that our review focuses on up‐to‐date interventions available from schools.

### 3.7 Search Methods and Sources

We propose a comprehensive search strategy to identify published and unpublished studies that potentially meet the eligibility criteria, following the guidelines provided by MacDonald et al. (2024). The following strategies will be used to search for studies.

#### 3.7.1 Electronic Searches

We identified two broad categories of keywords to use in the database search, related to the population and the intervention of interest. Since this search will be conducted as part of a larger scoping review of qualitative and quantitative studies on school dropout, we have not set any restrictions on the study designs for database search. Furthermore, no restrictions have been placed on the outcome type, due to the broad scope of the EGM, which also includes intermediate outcomes. The terms on population and intervention were identified from previous reviews on school dropout (Freeman and Simonsen 2015; Wang et al. 2024; Wilson et al. 2011) and from the titles and abstracts of seed articles (see Annex 4). The seed articles were identified by review authors to be geographically diverse and to include intermediate and/or long‐term outcomes.

We set up an initial search on ERIC (EBSCOhost) using multiple combinations of the identified keywords and controlled vocabulary provided by the database. The search was tested against the set of seed articles. The search string was adapted for the following databases. We used language and date of publication as limiters in searching all databases. The exact search strings for each database are available in Annex 3.

- Academic Search Premier (EBSCOhost).
- APA PsycInfo (EBSCOhost).
- Education Source (EBSCOhost).
- Education Resources Information Center‐ERIC (EBSCOhost).
- Scopus (Elsevier).
- Social Sciences Citation Index (Web of Science).
- Arts & Humanities Citation Index (Web of Science).

In addition, we planned to search in databases of dissertations and theses using the terms for intervention selected for the database search and terms related to the eligible research design to limit the search results (see Annex 3):

- ProQuest Dissertations & Theses Citation Index (Web of Science).
- Open Dissertations (EBSCOhost).

#### 3.7.2 Searching Other Resources

We will use the following complementary search methods to identify potentially eligible gray literature.

The following libraries, websites of organizations, and government agencies that publish or commission reviews and experimental studies will be hand‐searched:

- 3ie Systematic Review Database (https://www.3ieimpact.org/evidence‐hub/publications/systematic‐reviews).
- Campbell Collaboration (https://onlinelibrary.wiley.com/journal/18911803).
- Education Endowment Foundation (https://educationendowmentfoundation.org.uk/projects‐and‐evaluation/projects).
- EPPI‐Center (https://eppi.ioe.ac.uk/cms/Publications/tabid/56/Default.aspx).
- Institute of Education Sciences (https://ies.ed.gov/pubsearch/).
- National Dropout Prevention Center (https://dropoutprevention.org/resources/research‐reports/).
- Organization for Economic Co‐operation and Development (https://www.oecd.org/en/publications/reports.html?orderBy=mostRelevant&page=0).
- United Nations Educational, Scientific and Cultural Organization (https://www.unesco.org/en/tags/research).
- What Works Clearinghouse (https://ies.ed.gov/ncee/wwc/).
- World Bank Group (https://www.worldbank.org/en/research).

Conference proceedings are indexed in several electronic databases we will search (e.g., APA PsycInfo, Education Source, ERIC). Additionally, we will hand‐search the tables of contents or repositories of the following educational conferences from the past 5 years:

- American Educational Research Association (https://www.aera.net/Publications/Online‐Paper‐Repository/AERA‐Online‐Paper‐Repository).
- European Conference on Educational Research (https://eera‐ecer.de/conferences/ecer‐2024‐nicosia).
- Society for Research on Educational Effectiveness (https://www.sree.org/conferences).

We ran an explorative search in ERIC using the search string identified for this database (see Annex 3) and filtered the results to show the journals with the larger numbers of results. We selected the first top five journals to be hand‐searched by reviewing the tables of contents for the last 6 months: *Psychology in the Schools, Journal of School Health, Journal of Education for Students Placed at Risk, Journal of Educational Psychology, and Preventive School Failure*.

We identified previous reviews that evaluated the effects of programs to tackle school dropout or school dropout outcomes. To identify them (see the complete list in Annex 4), we searched the following journals devoted to evidence synthesis: *Campbell Systematic Reviews*, *Review of Educational Research*, and *Educational Research Reviews*. We also conducted a search in Google Scholar using various combinations of terms related to school dropout and experimental designs, screening the first 20–30 results for relevance. Eighteen systematic reviews were identified. We will conduct backward and forward citation chasing in the identified reviews. Backward citation chasing involves retrieving the records listed in the bibliography of one or more articles. Forward citation chasing involves locating records that have cited a specific article or a set of articles. This search will be conducted with the support of *Paperfetcher* (Pallath and Zhang 2022), an open‐source Python package and web app for hand searching in journals and citation chasing.

The first 100 hits of Google Scholar, searched for using different combinations of the following keywords, will be screened: “school dropout”, “school drop‐out”, “early school leaving”, “dropout prevention”, “dropout intervention”, “dropout compensation”, “high school graduation”, and “high school completion”.

We will use a snowball sampling approach to identify experts in school dropout research who are likely to be aware of relevant studies. After study selection, we will identify researchers whose names frequently appear in the included studies to inquire about any unpublished or ongoing research. Additionally, we will ask members of our Advisory Board to provide a list of experts in the field.

### 3.8 Analysis and Presentation

#### 3.8.1 Report Structure

The report structure will follow guidelines by White et al. (2020) and will include the following sections, tables, and figures:

Abstract

Plain Language Summary

Background

Figure 1: Logic model.

Objectives

Methods

Table 1: Reasons of exclusion in the full‐text review stage.

Table 2: Outcome domains, categories, and definitions.

Results

Table 3: Characteristics of the included studies.

Table 4: Results of critical appraisal.

Figure 2: PRISMA flowchart.

Figure 3: Snapshots of sections of the EGM.

Discussion

Conclusions

Annex 1 – Definitions of interventions.

Annex 2 – Definitions of outcomes.

Annex 3 – Search strings for database search.

Annex 4 – List of previous reviews.

Annex 5 – Codebook.

Annex 6 – Dataset.

#### 3.8.2 Filters for Presentation

In addition to programs and outcomes, several variables related to the PICOS framework will be coded and included in the online interactive EGM as filters to support stakeholders in selecting programs matching certain criteria of interest (see codebook in Annex 5). Among population characteristics, we plan to include the following filters: geographical area (Africa, Asia, Australia, Europe, North America, South America), grade level (early childhood education, elementary, middle, secondary), SES (low SES, average/high SES), urbanicity level (urban, suburban, rural), and program target population (students, teen parents, teachers, school leaders, parents). However, we will adapt them according to the information available in the included studies and on the basis of what will emerge from discussion with the stakeholders. From our previous experience in systematic reviews, we expect that a limited number of studies will provide information on SES and urbanicity, especially those conducted outside the United States. The following characteristics of the study design will also be included as filters: study design (meta‐analysis, RCT, QED); publication status (published, unpublished), study size (number of students), counterfactual (business as usual, alternative program), baseline equivalence for QEDs (demographic equivalence, pretest equivalence, no demographic equivalence, no pretest equivalence).

#### 3.8.3 Dependency

The unit of analysis is each study reporting the effects of a program. If a report covers multiple studies, each study will be treated as independent and coded in the data extraction phase. When multiple reports for a single study are retrieved, the most recent one will be the primary source for information and the older ones will be considered as duplicates. No preference will be made based on the publication status. If the primary source does not report all the characteristics needed for data extraction, the other reports will be consulted in search of the missing data.

### 3.9 Data Collection and Analysis

#### 3.9.1 Screening and Study Selection

After searching, all identified records will be uploaded on Covidence (https://www.covidence.org/), an online platform for reviews that supports a systematic and transparent process. Duplicate records will be automatically removed by Covidence. Any additional duplicates identified manually during the screening stage will also be addressed.

A two‐stage process will be used to select the eligible studies. In the first stage, the title and abstract of the retrieved studies will be screened, and then retained studies will be reviewed in full text. A tool to guide screening will be provided for the review team and will be constantly updated with explanations and examples during the process. Initial training will be conducted to practice screening on a weekly basis. In the first meeting, the titles and abstracts of 30 studies will be jointly screened by the team. Training will continue by screening 50 studies at a time, with disagreements resolved through meetings. After reaching a high level of agreement, studies will be independently screened by two team members, and disagreements will be jointly discussed and resolved by the review team. This iterative procedure will be followed until a 90% agreement among reviewers is reached. Then, studies will be screened by a single reviewer. If the agreement rate does not reach 90%, the screening will continue with two independent reviewers.

All retained studies will be independently reviewed in full text by two team members, based on the eligibility criteria. A tool with questions on eligibility criteria will be created, and training sessions will be conducted to guide the screening for this stage, following a similar approach to the one used for the screening stage. The review team will meet on a regular basis to discuss and resolve disagreements. When the team does not reach a consensus, an experienced reviewer will resolve the conflict. The study selection process will be carried out using *Covidence*. The tool has recently been equipped with a machine learning system that shows the records in order of relevance, based on the ongoing screening.

#### 3.9.2 Data Extraction and Management

Data extraction will be conducted in a structured, unambiguous form to collect relevant characteristics of the included studies. A draft codebook, which includes levels and definitions, has been developed, based on the PICOS framework, to extract relevant characteristics (see Annex 5). The codebook has been pre‐tested on two systematic reviews and two experimental studies, to make the necessary revisions. During the initial phase of data extraction, the codebook might undergo additional revisions by also collecting review proposals from stakeholders. These may involve adjustments to the characteristics to be coded, along with their corresponding levels and explanations. Initial training will be conducted on ten studies, which will be independently extracted by all coders, with disagreements discussed and jointly resolved. We will continue to double‐code studies until we reach 90% agreement on each characteristic. A coder will then extract data for the rest of the studies, with a second coder validating the responses. Any disagreements will be noted by the second coder and reviewed in meetings with the two coders, plus another author. During the data extraction phase, we will code characteristics that are not reported in the studies as missing. We will attempt to obtain the information from other reports of the same study and by contacting the authors. *MetaReviewer* (Polanin et al. 2023) will be used for data extraction.

#### 3.9.3 Tools for Assessing Risk of Bias/Study Quality of Included Reviews

The “Measurement Tool to Assess Systematic Reviews” (AMSTAR 2; Shea et al. 2017) will be used to conduct a critical appraisal of systematic reviews with meta‐analysis. A coder will assess the study quality of included reviews, with a second coder validating the responses. For experimental and quasi‐experimental studies, we will assess the quality of the included studies by coding the following study design characteristics (see also Annex 5): study design (meta‐analysis, RCT, QED); publication status (published, unpublished), study size (number of students), and counterfactual (business as usual, alternative program). Given the inclusion of QEDs with potential differences in the methodological quality, we will also code whether these designs demonstrate baseline equivalence between the treatment and control conditions in terms of demographics and outcomes evaluated (demographic equivalence, pretest equivalence, no demographic equivalence, and no pretest equivalence). We will use the critical value of 0.25 standard deviation (WWC 2022) to code the initial equivalence of the two groups for continuous variables and 0.10 for raw proportion dfference in demographics. The results of the risk of bias assessment for systematic reviews and primary studies will be discussed in the final report. For primary study quality assessment, the design quality characteristics will be used as filters in the interactive map. For systematic review quality assessment, the results of AMSTAR‐2 indicating the level of risk of bias will be included as a filter in the EGM.

#### 3.9.4 Methods for Mapping

EPPI Mapper will be used to generate an online interactive EGM. After data extraction, a JSON file will be exported from *MetaReviewer* and uploaded to EPPI Mapping to generate the map.

## Author Contributions

- Content: Marta Pellegrini, Carmen Pannone, Daniela Fadda, Laura Francesca Scalas, Giuliano Vivanet.
- EGM methods: Marta Pellegrini, Amanda Neitzel.
- Statistical analysis: Not applicable.
- Information retrieval: Daniela Fadda, Giuliano Vivanet.

## Conflicts of Interest

The authors declare no conflicts of interest.

## Preliminary Timeframe

We plan to submit the final EGM by December 2025.

## Plans for Updating the EGM

No plans for updating the EGM.

## Sources of Support

### 1 External Sources

This study was supported by Fondazione di Sardegna, Progetti biennali ‐ FdS 2022 (Grant number: F73C23001830007).

## Supporting information

Supporting information.
