# Supplementary material for: PROTOCOL: Prevention, Intervention, and Compensation Programs to Tackle School Dropout: An Evidence and Gap Map
Source: Campbell Syst Rev. 2025 Mar 13;21(1):e70032. doi: 10.1002/cl2.70032 (PMC11904426; doi:10.1002/cl2.70032)
Supplement: Supplementary file 1 — Supporting information. [file CL2-21-e70032-s001.docx]

**Annex 1: Definitions of interventions**

| **Prevention domain:**  Prevention aims to reduce the risk of school dropout before the problem starts, optimizing education opportunities offered to all students and removing any obstacles for school success throughout grade levels. |
| --- |
| **School/class structure**:  It includes class or grade reorganization, comparison of different sizes of school/class, and comparison of different types of school (e.g., freshman academies that support the transition between school levels). In fact, school and class size or type may have different effects on multiple school outcomes. (Rumberger, 2011; Wilson et al., 2011). |
| **Schoolwide approaches:**  Comprehensive strategies and initiatives implemented in educational institutions, typically at school level, to address various aspects of student learning, achievement, and well-being. These approaches aim to create a positive and inclusive school environment that supports the needs of all students, high levels of attendance and good behavior (Rumberger et al., 2017). |
| **Family engagement interventions:**  A broad range of activities involving families in their children’s education. They differ from measures in the Intervention domain in the fact that they apply indistinctly to every child. Examples are programs that educate parents on specific skills, or on various topics such as abuse or sexuality, identified as family strengthening (Chappell et al., 2015). |
| **Career-oriented programming:**  Programs and curricula that connect school with post-secondary school opportunities. They include college-focused and career-focused programs, and a variety of learning experiences planned to bridge the gap between theory and practice, such as apprenticeships and student internships (Chappell et al., 2015; Rumberger et al., 2017). |
| **School staff professional development:**  It encompasses all opportunities for professional development designed to equip school leaders, teachers, trainers, and other educational staff with the multiple roles and skills they need to acquire. For example, a professional development program might focus on understanding risk and protective factors for disengagement or early dropout, challenging gender stereotypes in teaching and learning, and utilizing a variety of learning approaches, tools, and formative assessment methods (Council Recommendation 2022/C 469/01, 2022; NDPC/N, 2022). |
| **Mentoring/counseling:**  A prolonged relationship between two or more individuals, wherein an older, nurturing, and more experienced person offers support to a younger individual as they navigate through life (Chappell et al., 2015). |
| **Community services:**  Programs involved in planning and carrying out a community service project with the specific goal of preventing early school leaving (Wilson et al., 2011). |
| **Academic strategies:**  They imply the adoption of teaching and learning strategies aimed at increasing the engagement and involvement of students in the learning process. Examples can include the adoption of strategies for (inter)active and experiential learning, individualized instruction, formative assessment, and educational technology (Council Recommendation 2022/C 469/01, 2022; NDPC/N, 2022). |
| **Out-of-school programs:**  They include extra-curricular activities such as recreational, sports, creative, performing arts, academic, or social activities happening in a safe, structured, and positive environment out of regular school hours (e.g., after school or during summertime) (Chappell et al., 2015). |
| **Early warning systems:**  Systems enabling the tracking of all students' progress and taking proactive measures when early indications of attendance, behavioral, linguistic, social, emotional, or academic difficulties arise (Council Recommendation 2022/C 469/01, 2022; Rumberger et al., 2017). |
| **Intervention domain:**  Intervention aims to avoid students dropping out of school by reacting to early warning signs and providing specific support for students at risk. These strategies target only students identified as at risk of dropping out of school. |
| **Skills training and behavioral interventions:**  They include interventions strengthening a wide set of behaviors, attitudes, and personal qualities so that students develop the ability to navigate their environment, work well with others, perform well, and achieve set goals. Examples of life skills include social skills, communication skills, problem solving skills, decision making skills and self-control, but also Cognitive Behavioral Therapy (CBT), conflict resolution, anger management, court advocacy, probation, transition, substance abuse prevention, and truancy prevention (Chappell et al., 2015; Lippman et al., 2015; Wilson et al., 2011). |
| **Career-oriented programming:**  Programs and curricula that connect school with post-secondary school opportunities. They include college-focused and career-focused programs, and a variety of learning experiences planned to bridge the gap between theory and practice, such as apprenticeships and student internships (Chappell et al., 2015; Rumberger et al., 2017). |
| **Multi-package services:** Large, comprehensive programs that often include academic, vocational, and case management (Wilson et al., 2011). |
| **Supplemental academic services:**  Strategies to raise students’ academic competences, including extra-time support, credit recovery, special curricula designed to help math or literacy development, homework assistance, and tutoring (Chappell et al., 2015; Rumberger, 2011; Wilson et al., 2011). |
| **Out-of-school programs:**  They include extra-curricular activities such as recreational, sports, creative, performing arts, or social activities happening in a safe, structured, and positive environment out of regular school hours (e.g., after school or during summertime) (Chappell et al., 2015). |
| **Community services:**  Programs involved in planning and carrying out a community service project with the specific goal of intervening in early school leaving (Wilson et al., 2011). |
| **Case management:**  It includes programs revolved around connecting students and families with appropriate services, such as health services, transportation assistance, residential living services, and financial support (Wilson et al., 2011). |
| **Mentoring/counseling:**  A personal relationship between one (or more) students identified as at-risk of dropping out and a caring and supportive adult, functioning as an advocate who can provide tailored assistance to address their academic, personal, and emotional requirements (Rumberger et al., 2017). |
| **Family engagement interventions:**  A broad range of activities involving families in their children’s education. They differ from measures in the Prevention domain in the fact that they apply only to specific cases identified as at-risk. Examples are programs identified as family therapy, aimed at enhancing dysfunctional patterns of family communication and interaction, or teen-parent support (Chappell et al., 2015). |
| **Compensation domain:**  Compensation is directed toward dropout outreach, assessment of student needs, and support with re-enrollment and persistence. |
| **Vocational Education and Training (VET):**  It is designed for participants to acquire knowledge, practical skills, and competencies necessary for a particular occupational field or trade. They can be either school-based programs or combined school- and work-based programs (i.e., with less than 75% of the curriculum presented in the school environment). The programs may include apprenticeship programs involving concurrent school-based and work-based training, as well as alternating periods of attendance at educational institutions and participation in work-based learning (European Education and Culture Executive Agency, Eurydice, 2014; Kis, 2020). |
| **Alternative schools:**  Educational institutions crafted to offer academic and additional services, such as behavioral support, to students whose needs are not adequately met in conventional schools. Typically catering to students who face challenges in mainstream educational settings, they include, for instance, online courses and GED preparation (Chappell et al., 2015; Wilson et al., 2011). |

**Annex 2: Definitions of outcomes**

| **Ultimate outcome domain:**  These outcomes are the direct evaluation of the planned long-term goal to be achieved, i.e., reducing school dropout rates and increase the school completion rates. |
| --- |
| **School dropout:**  Measure of students who leave education and training without an upper secondary qualification (Council recommendation 2022/C 469/01, 2022). |
| **School completion/graduation:**  School completion includes either a measure of high/secondary school graduates, a measure of students who achieved equivalent credentials (GED, I-VET, or similar certificate), or a measure of enrollers in post-secondary education (Rumberger, 2011; Wilson et al., 2011). |
| **Intermediate outcome domain:**  These outcomes refer to multiple types of individual risk factors/precursors of school dropout that could be seen (and addressed) in school-related contexts. They are grouped into educational performance, behaviors, and attitudes, as well as relational and social factors. |
| **Educational performance:**  This includes student academic performance, such as grades and test scores from standardized or researcher-made measures. It also includes educational persistence and attainment, meaning grade retention, academic track, and course failures (Rumberger, 2011; Wilson et al., 2011). |
| **Problem behaviors:**  Factors that reflect problem behaviors of students in and outside of school, such as school suspension, school expulsion, deviant behaviors, and substance use (Wilson, 2011). |
| **Attendance:**  School attendance is defined as a student being present in an accredited organized learning environment at any time during the school year (OECD, 2001). In this category we also included truancy and enrollment. |
| **Behaviors:**  Factors that reflect what students do in and outside of school, such as enrollment, attendance, truancy, school suspension, school expulsion, deviant behaviors, and substance use (Wilson, 2011). |
| **Attitudes:**  A wide range of psychological factors, such as sense of belonging, learning motivation, engagement, and well-being, but also values assigned to schooling, expectations, and goals. They also include factors related to the concept of self (e.g., self-perception, self-esteem, self-concept, self-efficacy, locus of control), since they are key components of achievement motivation and precursors of engagement (Rumberger, 2011). |
| **Relational and social factors:**  Factors related to interpersonal relationships include social skills, attitudes toward others, student-teacher interactions, peer relationships, and family relationships (Johansson, 2019). |

**Annex 3: Search strategy for school dropout evidence and gap map**

1. **ERIC (EBSCOhost):** https://search.ebscohost.com

| **#** | **Searches** | **Results** |
| --- | --- | --- |
|  | Population terms |  |
| S1 | TI ( school* or elementary or primary or secondary or kindergarten or grade*) N2 (student* or children)) or pupil* ) OR AB (school* or elementary or primary or secondary or kindergarten or grade*) N2 (student* or children)) or pupil* ) | 264,156 |
| S2 | DE "Elementary School Students" OR DE "Middle School Students" OR DE "Secondary School Students" OR DE "High School Students"  OR DE "Junior High School Students" OR DE "Adolescents" OR DE "Academic Education" OR DE "Early Childhood Education" OR DE "Elementary Secondary Education" OR DE "High School Equivalency Programs" OR DE "Kindergarten" | 492,250 |
| S3 | S1 OR S2 | 599,738 |
|  | Intervention terms |  |
| S4 | TI (school N3 dropout* OR student* N3 dropout* OR school N3 drop-out* OR student* N3 drop-out* OR school N3 drop out* OR dropout N3 rate* OR drop out N3 rate* OR drop-out N3 rate* OR student* N3 drop out* OR dropout N3 risk* OR drop-out N3 risk* OR drop out N3 risk* OR school N3 completion OR school N3 graduation OR graduation N3 rate* OR "high school graduate*" OR "high school diploma" OR "GED" OR "general education development" OR "school failure*" OR "dropout outcome*" OR "drop-out outcome*" OR "drop out outcome*" OR "dropping out" OR "school refusal*" OR "early school leav*" OR "early leav*" OR "school retention" OR "complet* school" OR "dropout prevention*" OR "dropout intervention*" OR "drop-out prevention*" OR "drop-out intervention*" OR "drop out prevention*" OR "drop out intervention*" OR "career academ*" OR "dropout program*" OR "drop-out program*" OR "drop out program*" OR "early warning system*" OR "early warning intervention*" OR "early warning indicator*" OR "dropout potential" OR "drop out potential"  OR "drop-out potential")  OR  AB (school N3 dropout* OR student* N3 dropout* OR school N3 drop-out* OR student* N3 drop-out* OR school N3 drop out* OR dropout N3 rate* OR drop out N3 rate* OR drop-out N3 rate* OR student* N3 drop out* OR dropout N3 risk* OR drop-out N3 risk* OR drop out N3 risk* OR school N3 completion OR school N3 graduation OR graduation N3 rate* OR "high school graduate*" OR "high school diploma" OR "GED" OR "general education development" OR "school failure*" OR "dropout outcome*" OR "drop-out outcome*" OR "drop out outcome*" OR "dropping out" OR "school refusal*" OR "early school leav*" OR "early leav*" OR "school retention" OR "complet* school" OR "dropout prevention*" OR "dropout intervention*" OR "drop-out prevention*" OR "drop-out intervention*" OR "drop out prevention*" OR "drop out intervention*" OR "career academ*" OR "dropout program*" OR "drop-out program*" OR "drop out program*" OR "early warning system*" OR "early warning intervention*" OR "early warning indicator*" OR "dropout potential" OR "drop out potential"  OR "drop-out potential") | 29,749 |
| S5 | DE "At Risk Students" OR DE "Academic Failure" OR DE "Dropout Prevention" OR DE "Dropout Programs" OR DE "High School Equivalency Programs" OR DE "Out of School Youth" OR DE "Persistence" OR DE "Reentry Students" OR DE "Retraining" OR DE "Stopouts" OR DE "Potential Dropouts" OR DE "Student Attrition" OR DE "Enrollment" OR DE "Academic Persistence" OR DE "Dropout Rate" OR DE "Dropout Research" OR DE "Dropouts" OR DE "Withdrawal (Education)" | 59,842 |
| S6 | S4 OR S5 | 75,579 |
| S7 | S3 AND S6 | 33,135 |
| S8 | S7 AND Publication Date (20110101-) AND Language (English) | 14,750 |

1. **Education Source (EBSCOhost):** https://search.ebscohost.com

| **#** | **Searches** | | **Results** |
| --- | --- | --- | --- |
|  | Population terms | |  |
| S1 | TI ( school* or elementary or primary or secondary or kindergarten or grade*) N2 (student* or children)) or pupil* ) OR AB (school* or elementary or primary or secondary or kindergarten or grade*) N2 (student* or children)) or pupil* ) | 385,299 | |
| S2 | DE "At-risk students" OR DE "Education students" OR DE "Re-entry students" OR DE "School children" OR DE "Early childhood education" OR DE "Elementary education" OR DE "Middle school education" OR DE "Secondary education" OR DE "Kindergarten" | | 150,485 |
| S3 | S1 OR S2 | | 466,231 |
|  | Intervention terms | |  |
| S4 | TI (school N3 dropout* OR student* N3 dropout* OR school N3 drop-out* OR student* N3 drop-out* OR school N3 drop out* OR dropout N3 rate* OR drop out N3 rate* OR drop-out N3 rate* OR student* N3 drop out* OR dropout N3 risk* OR drop-out N3 risk* OR drop out N3 risk* OR school N3 completion OR school N3 graduation OR graduation N3 rate* OR "high school graduate*" OR "high school diploma" OR "GED" OR "general education development" OR "school failure*" OR "dropout outcome*" OR "drop-out outcome*" OR "drop out outcome*" OR "dropping out" OR "school refusal*" OR "early school leav*" OR "early leav*" OR "school retention" OR "complet* school" OR "dropout prevention*" OR "dropout intervention*" OR "drop-out prevention*" OR "drop-out intervention*" OR "drop out prevention*" OR "drop out intervention*" OR "career academ*" OR "dropout program*" OR "drop-out program*" OR "drop out program*" OR "early warning system*" OR "early warning intervention*" OR "early warning indicator*" OR "dropout potential" OR "drop out potential"  OR "drop-out potential")  OR  AB (school N3 dropout* OR student* N3 dropout* OR school N3 drop-out* OR student* N3 drop-out* OR school N3 drop out* OR dropout N3 rate* OR drop out N3 rate* OR drop-out N3 rate* OR student* N3 drop out* OR dropout N3 risk* OR drop-out N3 risk* OR drop out N3 risk* OR school N3 completion OR school N3 graduation OR graduation N3 rate* OR "high school graduate*" OR "high school diploma" OR "GED" OR "general education development" OR "school failure*" OR "dropout outcome*" OR "drop-out outcome*" OR "drop out outcome*" OR "dropping out" OR "school refusal*" OR "early school leav*" OR "early leav*" OR "school retention" OR "complet* school" OR "dropout prevention*" OR "dropout intervention*" OR "drop-out prevention*" OR "drop-out intervention*" OR "drop out prevention*" OR "drop out intervention*" OR "career academ*" OR "dropout program*" OR "drop-out program*" OR "drop out program*" OR "early warning system*" OR "early warning intervention*" OR "early warning indicator*" OR "dropout potential" OR "drop out potential"  OR "drop-out potential") | | 23,196 |
| S5 | DE "Evening & continuation school students" OR DE "High school equivalency certificates" OR DE "Elementary school dropouts" OR DE "High school dropouts" OR DE "Junior high school dropouts" OR DE "Middle school dropouts" OR DE "Vocational school dropouts" OR DE "Dropout rates (Education)" OR DE "Graduation rate" OR DE "Re-entry students" OR DE "School failure" OR DE "Stopouts" OR DE "Student attrition" OR DE "School dropout prevention" OR DE "School dropout recovery programs" OR DE "School dropouts" OR DE "Services for school dropouts" | | 13,273 |
| S6 | S4 OR S5 | | 29,754 |
| S7 | S3 AND S6 | | 8,268 |
| S8 | S7 AND Publication Date (20110101-) AND Language (English) | | 2,953 |

1. **APA PsycInfo (EBSCOhost):** https://search.ebscohost.com

| **#** | **Searches** | | **Results** |
| --- | --- | --- | --- |
|  | Population terms | |  |
| S1 | TI ( school* or elementary or primary or secondary or kindergarten or grade*) N2 (student* or children)) or pupil* ) OR AB ( school* or  elementary or primary or secondary or kindergarten or grade*) N2 (student* or children)) or pupil* ) | 233,205 | |
| S2 | DE "Elementary School Students" OR DE "High School Students" OR DE "Junior High School Students" OR DE "Kindergarten Students" OR DE "Middle School Students" OR DE "Reentry Students" OR DE "Elementary Education" OR DE "High School Education" OR DE "Middle School Education" | | 108,615 |
| S3 | S1 OR S2 | | 271,277 |
|  | Intervention terms | |  |
| S4 | TI (school N3 dropout* OR student* N3 dropout* OR school N3 drop-out* OR student* N3 drop-out* OR school N3 drop out* OR dropout N3 rate* OR drop out N3 rate* OR drop-out N3 rate* OR student* N3 drop out* OR dropout N3 risk* OR drop-out N3 risk* OR drop out N3 risk* OR school N3 completion OR school N3 graduation OR graduation N3 rate* OR "high school graduate*" OR "high school diploma" OR "GED" OR "general education development" OR "school failure*" OR "dropout outcome*" OR "drop-out outcome*" OR "drop out outcome*" OR "dropping out" OR "school refusal*" OR "early school leav*" OR "early leav*" OR "school retention" OR "complet* school" OR "dropout prevention*" OR "dropout intervention*" OR "drop-out prevention*" OR "drop-out intervention*" OR "drop out prevention*" OR "drop out intervention*" OR "career academ*" OR "dropout program*" OR "drop-out program*" OR "drop out program*" OR "early warning system*" OR "early warning intervention*" OR "early warning indicator*" OR "dropout potential" OR "drop out potential"  OR "drop-out potential")  OR  AB (school N3 dropout* OR student* N3 dropout* OR school N3 drop-out* OR student* N3 drop-out* OR school N3 drop out* OR dropout N3 rate* OR drop out N3 rate* OR drop-out N3 rate* OR student* N3 drop out* OR dropout N3 risk* OR drop-out N3 risk* OR drop out N3 risk* OR school N3 completion OR school N3 graduation OR graduation N3 rate* OR "high school graduate*" OR "high school diploma" OR "GED" OR "general education development" OR "school failure*" OR "dropout outcome*" OR "drop-out outcome*" OR "drop out outcome*" OR "dropping out" OR "school refusal*" OR "early school leav*" OR "early leav*" OR "school retention" OR "complet* school" OR "dropout prevention*" OR "dropout intervention*" OR "drop-out prevention*" OR "drop-out intervention*" OR "drop out prevention*" OR "drop out intervention*" OR "career academ*" OR "dropout program*" OR "drop-out program*" OR "drop out program*" OR "early warning system*" OR "early warning intervention*" OR "early warning indicator*" OR "dropout potential" OR "drop out potential"  OR "drop-out potential") | | 19,824 |
| S5 | DE "Potential Dropouts" OR DE "Educational Attainment Level" OR DE "High School Graduates" OR DE "Reentry Students" OR DE "School Refusal" OR DE "Student Attrition" OR DE "School Graduation" OR DE "School Dropout" | | 12,768 |
| S6 | S4 OR S5 | | 28,607 |
| S7 | S3 AND S6 | | 7,456 |
| S8 | S7 AND Publication Date (20110101-) AND Language (English) | | 3,688 |

1. **Academic Search Premier (EBSCOhost):** https://search.ebscohost.com

| **#** | **Searches** | | **Results** |
| --- | --- | --- | --- |
|  | Population terms | |  |
| S1 | TI ( school* or elementary or primary or secondary or kindergarten or grade*) N2 (student* or children)) or pupil* ) OR AB ( school* or  elementary or primary or secondary or kindergarten or grade*) N2 (student* or children)) or pupil* ) | 312,481 | |
| S2 | DE "MIDDLE school students" OR DE "MIDDLE school education" OR DE "KINDERGARTEN" OR DE "EARLY childhood education" OR DE "KINDERGARTEN children" OR DE "PRIMARY schools" OR DE "ELEMENTARY education" OR DE "RECEPTION classes" OR DE "ELEMENTARY schools" OR DE "PRIMARY education" OR DE "SECONDARY school students" OR DE "HIGH school students" OR DE "SECONDARY education" OR DE "SECONDARY schools" | | 100,992 |
| S3 | S1 OR S2 | | 368,081 |
|  | Intervention terms | |  |
| S4 | TI (school N3 dropout* OR student* N3 dropout* OR school N3 drop-out* OR student* N3 drop-out* OR school N3 drop out* OR dropout N3 rate* OR drop out N3 rate* OR drop-out N3 rate* OR student* N3 drop out* OR dropout N3 risk* OR drop-out N3 risk* OR drop out N3 risk* OR school N3 completion OR school N3 graduation OR graduation N3 rate* OR "high school graduate*" OR "high school diploma" OR "GED" OR "general education development" OR "school failure*" OR "dropout outcome*" OR "drop-out outcome*" OR "drop out outcome*" OR "dropping out" OR "school refusal*" OR "early school leav*" OR "early leav*" OR "school retention" OR "complet* school" OR "dropout prevention*" OR "dropout intervention*" OR "drop-out prevention*" OR "drop-out intervention*" OR "drop out prevention*" OR "drop out intervention*" OR "career academ*" OR "dropout program*" OR "drop-out program*" OR "drop out program*" OR "early warning system*" OR "early warning intervention*" OR "early warning indicator*" OR "dropout potential" OR "drop out potential"  OR "drop-out potential")  OR  AB (school N3 dropout* OR student* N3 dropout* OR school N3 drop-out* OR student* N3 drop-out* OR school N3 drop out* OR dropout N3 rate* OR drop out N3 rate* OR drop-out N3 rate* OR student* N3 drop out* OR dropout N3 risk* OR drop-out N3 risk* OR drop out N3 risk* OR school N3 completion OR school N3 graduation OR graduation N3 rate* OR "high school graduate*" OR "high school diploma" OR "GED" OR "general education development" OR "school failure*" OR "dropout outcome*" OR "drop-out outcome*" OR "drop out outcome*" OR "dropping out" OR "school refusal*" OR "early school leav*" OR "early leav*" OR "school retention" OR "complet* school" OR "dropout prevention*" OR "dropout intervention*" OR "drop-out prevention*" OR "drop-out intervention*" OR "drop out prevention*" OR "drop out intervention*" OR "career academ*" OR "dropout program*" OR "drop-out program*" OR "drop out program*" OR "early warning system*" OR "early warning intervention*" OR "early warning indicator*" OR "dropout potential" OR "drop out potential"  OR "drop-out potential") | | 39,876 |
| S5 | DE "HIGH school graduation rates" OR DE "HIGH school dropout rates" OR DE "ELEMENTARY school dropouts" OR DE "HIGH school dropouts" OR DE "JUNIOR high school dropouts" OR DE "MIDDLE school dropouts" OR DE "SCHOOL failure" OR DE "STOPOUTS" OR DE "SCHOOL dropout recovery programs" OR DE "HIGH school equivalency certificates" OR DE "SCHOOL dropout prevention" OR DE "EVENING & continuation school students" OR DE "RE-entry students" | | 4,088 |
| S6 | S4 OR S5 | | 40,271 |
| S7 | S3 AND S6 | | 6,251 |
| S8 | S7 AND Publication Date (20110101-) AND Language (English) | | 3,026 |

1. **Social Sciences Citation Index (Web of Science):** www.webofscience.com

| **#** | **Searches** | | **Results** |
| --- | --- | --- | --- |
|  | Population terms | |  |
| S1 | (TI=(school* NEAR/2 student* OR school* NEAR/2 children OR school* NEAR/2 pupil* OR elementary NEAR/2 student* OR elementary NEAR/2 children OR elementary NEAR/2 pupil* OR primary NEAR/2 student* OR primary NEAR/2 children OR primary NEAR/2 pupil* OR secondary NEAR/2 student* OR secondary NEAR/2 children OR secondary NEAR/2 pupil* OR kindergarten NEAR/2 student* OR kindergarten NEAR/2 children OR kindergarten NEAR/2 pupil* OR grade* NEAR/2 student* OR grade* NEAR/2 children OR grade* NEAR/2 pupil*)) OR (AB=(school* NEAR/2 student* OR school* NEAR/2 children OR school* NEAR/2 pupil* OR elementary NEAR/2 student* OR elementary NEAR/2 children OR elementary NEAR/2 pupil* OR primary NEAR/2 student* OR primary NEAR/2 children OR primary NEAR/2 pupil* OR secondary NEAR/2 student* OR secondary NEAR/2 children OR secondary NEAR/2 pupil* OR kindergarten NEAR/2 student* OR kindergarten NEAR/2 children OR kindergarten NEAR/2 pupil* OR grade* NEAR/2 student* OR grade* NEAR/2 children OR grade* NEAR/2 pupil*)) | 128,878 | |
|  | Intervention terms | |  |
| S2 | (TI=(school NEAR/3 dropout* OR student* NEAR/3 dropout* OR school NEAR/3 drop-out* OR student* NEAR/3 drop-out* OR school NEAR/3 drop out* OR dropout NEAR/3 rate* OR drop-out NEAR/3 rate* OR drop out NEAR/3 rate* OR student* NEAR/3 drop out* OR dropout NEAR/3 risk* OR drop-out NEAR/3 risk* OR drop out NEAR/3 risk* OR school NEAR/3 completion OR school NEAR/3 graduation OR graduation NEAR/3 rate* OR "high school graduate*" OR "high school diploma" OR "GED" OR "general education development" OR "school failure*" OR "dropout outcome*" OR "drop-out outcome*" OR "drop out outcome*" OR "dropping out" OR "school refusal*" OR "early school leav*" OR "early leav*" OR "school retention" OR "complet* school" OR "dropout prevention*" OR "dropout intervention*" OR "drop-out prevention*" OR "drop-out intervention*" OR "drop out prevention*" OR "drop out intervention*" OR "career academ*" OR "dropout program*" OR "drop-out program*" OR "drop out program*" OR "early warning system*" OR "early warning intervention*" OR "early warning indicator*" OR "dropout potential" OR "drop-out potential" OR "drop out potential"))  OR  (TS=(school NEAR/3 dropout* OR student* NEAR/3 dropout* OR school NEAR/3 drop-out* OR student* NEAR/3 drop-out* OR school NEAR/3 drop out* OR dropout NEAR/3 rate* OR drop-out NEAR/3 rate* OR drop out NEAR/3 rate* OR student* NEAR/3 drop out* OR dropout NEAR/3 risk* OR drop-out NEAR/3 risk* OR drop out NEAR/3 risk* OR school NEAR/3 completion OR school NEAR/3 graduation OR graduation NEAR/3 rate* OR "high school graduate*" OR "high school diploma" OR "GED" OR "general education development" OR "school failure*" OR "dropout outcome*" OR "drop-out outcome*" OR "drop out outcome*" OR "dropping out" OR "school refusal*" OR "early school leav*" OR "early leav*" OR "school retention" OR "complet* school" OR "dropout prevention*" OR "dropout intervention*" OR "drop-out prevention*" OR "drop-out intervention*" OR "drop out prevention*" OR "drop out intervention*" OR "career academ*" OR "dropout program*" OR "drop-out program*" OR "drop out program*" OR "early warning system*" OR "early warning intervention*" OR "early warning indicator*" OR "dropout potential" OR "drop-out potential" OR "drop out potential")) | | 16,671 |
| S3 | TS=(school NEAR/3 dropout* or school NEAR/3 failure* or "early school leav*") | | 3,630 |
| S4 | S2 OR S3 | | 17,216 |
| S5 | S1 AND S4 | | 2,142 |
| S6 | S5 AND Publication Date (20110101-) AND LA== (“ENGLISH”) | | 383 |

1. **Arts & Humanities Citation Index (Web of Science):** www.webofscience.com

| **#** | **Searches** | **Results** |
| --- | --- | --- |
|  | Intervention terms |  |
| S1 | (TI=(school NEAR/3 dropout* OR student* NEAR/3 dropout* OR school NEAR/3 drop-out* OR student* NEAR/3 drop-out* OR school NEAR/3 drop out* OR dropout NEAR/3 rate* OR drop-out NEAR/3 rate* OR drop out NEAR/3 rate* OR student* NEAR/3 drop out* OR dropout NEAR/3 risk* OR drop-out NEAR/3 risk* OR drop out NEAR/3 risk* OR school NEAR/3 completion OR school NEAR/3 graduation OR graduation NEAR/3 rate* OR "high school graduate*" OR "high school diploma" OR "GED" OR "general education development" OR "school failure*" OR "dropout outcome*" OR "drop-out outcome*" OR "drop out outcome*" OR "dropping out" OR "school refusal*" OR "early school leav*" OR "early leav*" OR "school retention" OR "complet* school" OR "dropout prevention*" OR "dropout intervention*" OR "drop-out prevention*" OR "drop-out intervention*" OR "drop out prevention*" OR "drop out intervention*" OR "career academ*" OR "dropout program*" OR "drop-out program*" OR "drop out program*" OR "early warning system*" OR "early warning intervention*" OR "early warning indicator*" OR "dropout potential" OR "drop-out potential" OR "drop out potential"))  OR  (TS=(school NEAR/3 dropout* OR student* NEAR/3 dropout* OR school NEAR/3 drop-out* OR student* NEAR/3 drop-out* OR school NEAR/3 drop out* OR dropout NEAR/3 rate* OR drop-out NEAR/3 rate* OR drop out NEAR/3 rate* OR student* NEAR/3 drop out* OR dropout NEAR/3 risk* OR drop-out NEAR/3 risk* OR drop out NEAR/3 risk* OR school NEAR/3 completion OR school NEAR/3 graduation OR graduation NEAR/3 rate* OR "high school graduate*" OR "high school diploma" OR "GED" OR "general education development" OR "school failure*" OR "dropout outcome*" OR "drop-out outcome*" OR "drop out outcome*" OR "dropping out" OR "school refusal*" OR "early school leav*" OR "early leav*" OR "school retention" OR "complet* school" OR "dropout prevention*" OR "dropout intervention*" OR "drop-out prevention*" OR "drop-out intervention*" OR "drop out prevention*" OR "drop out intervention*" OR "career academ*" OR "dropout program*" OR "drop-out program*" OR "drop out program*" OR "early warning system*" OR "early warning intervention*" OR "early warning indicator*" OR "dropout potential" OR "drop-out potential" OR "drop out potential")) | 222 |
| S2 | TS=(school NEAR/3 dropout* or school NEAR/3 failure* or "early school leav*") | 74 |
| S3 | S1 OR S2 | 254 |
| S4 | S3 AND Publication Date (20110101-) AND LA== (“ENGLISH”) | 140 |

1. **Scopus:** www.scopus.com

| **#** | **Searches** | | **Results** |
| --- | --- | --- | --- |
|  | Population terms | |  |
| S1 | (TITLE-ABS-KEY((school* OR student* OR children OR pupil*) W/3 (elementary OR primary OR secondary OR kindergarten OR middle OR grade*))) | 344,467 | |
|  | Intervention terms | |  |
| S2 | (TITLE-ABS-KEY("school dropout*" OR "student* dropout*" OR "school drop-out*" OR "student* drop-out*" OR "school drop out*" OR "dropout rate*" OR "drop-out rate*" OR "drop out rate*" OR "student* drop out*" OR "dropout risk*" OR "drop-out risk*" OR "drop out risk*" OR "school completion" OR "school graduation" OR "graduation rate*" OR "high school graduate*" OR "high school diploma" OR "GED" OR "general education development" OR "school failure*" OR "dropout outcome*" OR "drop-out outcome*" OR "drop out outcome*" OR "dropping out" OR "school refusal*" OR "early school leav*" OR "early leav*" OR "school retention" OR "complet* school" OR "dropout prevention*" OR "dropout intervention*" OR "drop-out prevention*" OR "drop-out intervention*" OR "drop out prevention*" OR "drop out intervention*" OR "career academ*" OR "dropout program*" OR "drop-out program*" OR "drop out program*" OR "early warning system*" OR "early warning intervention*" OR "early warning indicator*" OR "dropout potential" OR "drop-out potential" OR "drop out potential")) | | 55,667 |
| S3 | S1 AND S2 | | 3,918 |
| S4 | S3 AND Publication Date (20110101-) AND Language (English) | | 2,497 |

1. **ProQuest Dissertations & Theses Citation Index (via Web of Science):** www.webofscience.com

| S1 | (TI=(school N3 dropout* OR student* N3 dropout* OR school N3 drop-out* OR student* N3 drop-out* OR school N3 drop out* OR dropout N3 rate* OR drop out N3 rate* OR drop-out N3 rate* OR student* N3 drop out* OR dropout N3 risk* OR drop-out N3 risk* OR drop out N3 risk* OR school N3 completion OR school N3 graduation OR graduation N3 rate* OR "high school graduate*" OR "high school diploma" OR "GED" OR "general education development" OR "school failure*" OR "dropout outcome*" OR "drop-out outcome*" OR "drop out outcome*" OR "dropping out" OR "school refusal*" OR "early school leav*" OR "early leav*" OR "school retention" OR "complet* school" OR "dropout prevention*" OR "dropout intervention*" OR "drop-out prevention*" OR "drop-out intervention*" OR "drop out prevention*" OR "drop out intervention*" OR "career academ*" OR "dropout program*" OR "drop-out program*" OR "drop out program*" OR "early warning system*" OR "early warning intervention*" OR "early warning indicator*" OR "dropout potential" OR "drop out potential" OR "drop-out potential"))  OR  (AB=(school N3 dropout* OR student* N3 dropout* OR school N3 drop-out* OR student* N3 drop-out* OR school N3 drop out* OR dropout N3 rate* OR drop out N3 rate* OR drop-out N3 rate* OR student* N3 drop out* OR dropout N3 risk* OR drop-out N3 risk* OR drop out N3 risk* OR school N3 completion OR school N3 graduation OR graduation N3 rate* OR "high school graduate*" OR "high school diploma" OR "GED" OR "general education development" OR "school failure*" OR "dropout outcome*" OR "drop-out outcome*" OR "drop out outcome*" OR "dropping out" OR "school refusal*" OR "early school leav*" OR "early leav*" OR "school retention" OR "complet* school" OR "dropout prevention*" OR "dropout intervention*" OR "drop-out prevention*" OR "drop-out intervention*" OR "drop out prevention*" OR "drop out intervention*" OR "career academ*" OR "dropout program*" OR "drop-out program*" OR "drop out program*" OR "early warning system*" OR "early warning intervention*" OR "early warning indicator*" OR "dropout potential" OR "drop out potential" OR "drop-out potential")) | 9,834 |
| --- | --- | --- |
| S2 | (TI=("matched stud*" OR "quasi-experiment*" OR "quasi experiment*" OR QED RCT OR “randomized controlled trial*” OR “randomised controlled trial*” OR experiment* OR random* OR "non-random" “non random*” OR "effectiveness stud*" OR "efficacy stud*" OR "effectiveness trial*" OR "efficacy trial*" OR "impact evaluation*" OR “natural experiment*” OR “effect size” OR “comparison group*” OR “control group*” OR “matched group*” OR “matched control group*”)) OR AB=("matched stud*" OR "quasi-experiment*" OR "quasi experiment*" OR QED RCT OR “randomized controlled trial*” OR “randomised controlled trial*” OR experiment* OR random* OR "non-random" “non random*” OR "effectiveness stud*" OR "efficacy stud*" OR "effectiveness trial*" OR "efficacy trial*" OR "impact evaluation*" OR “natural experiment*” OR “effect size” OR “comparison group*” OR “control group*” OR “matched group*” OR “matched control group*”) | 954,830 |
| S3 | S1 AND S2 | 1,235 |
| S4 | S1 AND S2 AND Publication Date (20110101-) AND Language (English) | 1,082 |

1. **Open Dissertations (EBSCOhost):** www.webofscience.com

| S1 | TI ( school N3 dropout* OR student* N3 dropout* OR school N3 drop- out* OR student* N3 drop-out* OR school N3 drop out* OR dropout  N3 rate* OR drop out N3 rate* OR drop-out N3 rate* OR student* N3 drop out* OR dropout N3 risk* OR drop-out N3 risk* OR drop out N3 risk* OR school N3 completion OR school N3 graduation OR graduation N3 rate* OR "high school graduate*" OR "high school diploma" OR "GED" OR "general education development" OR "school failure*" OR "dropout outcome*" OR "drop-out outcome*" OR "drop out outcome*" OR "dropping out" OR "school refusal*" OR "early school leav*" OR "early leav*" OR "school retention" OR "complet* school" OR "dropout prevention*" OR "dropout intervention*" OR "drop-out prevention*" OR "drop-out intervention*" OR "drop out prevention*" OR "drop out intervention*" OR "career academ*" OR "dropout program*" OR "drop-out program*" OR "drop out program*" OR "early warning system*" OR "early warning intervention*" OR "early warning indicator*" OR "dropout potential" OR "drop out potential" OR "drop- out potential" ) OR AB ( school N3 dropout* OR student* N3 dropout* OR school N3 drop-out* OR student* N3 drop-out* OR school N3 drop out* OR dropout N3 rate* OR drop out N3 rate* OR drop-out N3 rate* OR student* N3 drop out* OR dropout N3 risk* OR drop-out N3 risk* OR drop out N3 risk* OR school N3 completion OR school N3 graduation OR graduation N3 rate* OR "high school graduate*" OR "high school diploma" OR "GED" OR "general education development" OR "school failure*" OR "dropout outcome*" OR "drop-out outcome*" OR "drop out outcome*" OR "dropping out" OR "school refusal*" OR "early school leav*" OR "early leav*" OR "school retention" OR "complet* school" OR "dropout prevention*" OR "dropout intervention*" OR "drop-out prevention*" OR "drop-out intervention*" OR "drop out prevention*" OR "drop out intervention*" OR "career academ*" OR "dropout program*" OR "drop-out program*" OR "drop out program*" OR "early warning system*" OR "early warning intervention*" OR "early warning indicator*" OR "dropout potential" OR "drop out potential" OR "drop-out potential" ) | 3,311 |
| --- | --- | --- |
| S2 | TI ( "matched stud*" OR "quasi-experiment*" OR "quasi experiment*" OR QED RCT OR “randomized controlled trial*” OR “randomised  controlled trial*” OR experiment* OR random* OR "non-random" “non random*” OR "effectiveness stud*" OR "efficacy stud*" OR "effectiveness trial*" OR "efficacy trial*" OR "impact evaluation*" OR “natural experiment*” OR “effect size” OR “comparison group*” OR “control group*” OR “matched group*” OR “matched control group*”) ) OR AB ( "matched stud*" OR "quasi-experiment*" OR "quasi experiment*" OR QED RCT OR “randomized controlled trial*” OR “randomised controlled trial*” OR experiment* OR random* OR "non- random" “non random*” OR "effectiveness stud*" OR "efficacy stud*" OR "effectiveness trial*" OR "efficacy trial*" OR "impact evaluation*" OR “natural experiment*” OR “effect size” OR “comparison group*” OR “control group*” OR “matched group*” OR “matched control group*”) ) | 215,581 |
| S3 | S1 AND S2 | 469 |
| S4 | S1 AND S2 AND Publication Date (20110101-) AND Language (English) | 338 |

**Annex 4: List of previous reviews**

1. Bartlett, D. (2017). *Systematic Review of High School Dropout Prevention Programs*. [Clinical Research Paper, St. Catherine University]. Sophia, the St. Catherine University repository. <https://sophia.stkate.edu/msw_papers/706>
2. Chappell, S., O’Connor, P., Withington, C., & Stegelin, D. (2015). *A Meta-Analysis of Dropout Prevention Outcomes and Strategies*. Clemson, SC: National Dropout Prevention Center/Network at Clemson University. The Center for Educational Partnerships at Old Dominion University and the National Dropout Prevention Center/Network at Clemson University. <https://dropoutprevention.org/wp-content/uploads/2022/11/A-Meta-Analysis-of-DOP-Outcomes-and-Strategies-Chappell-et-al-2015.pdf>
3. Freeman, J., & Simonsen, B. (2015). Examining the impact of policy and practice interventions on high school dropout and school completion rates: A systematic review of the literature.*Review of Educational Research*, *85*(2), 205–248.  <https://journals.sagepub.com/doi/abs/10.3102/0034654314554431>
4. Iachini, A. L., Brown, E. L., Ball, A., Gibson, J. E., & Lize, S. E. (2015). School mental health early interventions and academic outcomes for at-risk high school students: A meta-analysis. *Advances in School Mental Health Promotion*, *8*(3), 156–175. <https://doi.org/10.1080/1754730X.2015.1044252>
5. Johansson, B. (2019). *Dropping Out of School - A Systematic and Integrative Research Review on Risk Factors and Interventions*. Örebro University. <http://urn.kb.se/resolve?urn=urn:nbn:se:oru:diva-77853>
6. Klima, T., Miller, M., & Nunlist, C. (2009). *What works? Targeted truancy and dropout programs in middle and high school*. Olympia: Washington State Institute for Public Policy, No. 09-06-2201. <https://www.wsipp.wa.gov/ReportFile/1045/Wsipp_What-Works-Targeted-Truancy-and-Dropout-Programs-in-Middle-and-High-School_Full-Report.pdf>
7. Lehr, C. A., Hansen, A., Sinclair, M. F., & Christenson, S. L. (2003). Moving beyond dropout toward school completion: An integrative review of data-based interventions. *School Psychology Review*, *32*, 342–364.

<https://doi.org/10.1080/02796015.2003.12086205>

1. Maynard, B. R., Brendel, K. E., Bulanda, J. J., Heyne, D., Thompson, A. M., & Pigott, T. D. (2015). Psychosocial interventions for school refusal with primary and secondary school students: A systematic review. *Campbell Systematic Reviews,* *11*(1), 1–76. <https://doi.org/10.4073/csr.2015.12>
2. Petrosino, A., Morgan, C., Fronius, T. A., Tanner-Smith, E. E., & Boruch, R. F. (2012). Interventions in developing nations for improving primary and secondary school enrollment of children: A systematic review. *Campbell Systematic Reviews*, *8*, i-192. <https://doi.org/10.4073/csr.2012.19>.
3. Prevatt, F., & Kelly, F. D. (2003). Dropping out of School: A Review of Intervention Programs. *Journal of School Psychology*, *41*(5), 377–395.

<https://doi.org/10.1016/S0022-4405(03)00087-6>

1. Rumberger, R., Addis, H., Allensworth, E., Balfanz, R., Bruch, J., Dillon, E., Duardo, D., Dynarski, M., Furgeson, J., Jayanthi, M., Newman-Gonchar, R., Place, K., & Tuttle, C. (2017). Preventing drop-out in secondary schools (NCEE 2017-4028). Washington, DC: National Center for Education Evaluation and Regional Assistance (NCEE), Institute of Education Sciences, U.S. Department of Education. <https://ies.ed.gov/ncee/wwc/docs/practiceguide/wwc_dropout_092617.pdf>
2. Strom, R. E., & Boster, F. J. (2007). Dropping out of High School: A Meta-Analysis Assessing the Effect of Messages in the Home and in School. *Communication Education,* *56*(4), 433–452.

<https://doi.org/10.1080/03634520701413804>

1. Tanner-Smith, E. E., & Wilson, S. J. (2013). A Meta-analysis of the Effects of Dropout Prevention Programs on School Absenteeism. *Prevention Science, 14*, 468–478. <https://doi.org/10.1007/s11121-012-0330-1>
2. Valdebenito, S., Eisner, M., Farrington, D. P., Ttofi, M. M., & Sutherland, A. (2018). School-based interventions for reducing disciplinary school exclusion: a systematic review. *Campbell Systematic Reviews*, *14*, i-216. <https://doi.org/10.4073/csr.2018.1>
3. Wang, Q., Hsiao, Y.-Y., Hushman, C., & Armstrong, J. (2024). The effectiveness of dropout intervention programs among K-12 students: A meta-analysis. *Journal of Education for Students Placed at Risk.* Advance online publication. <https://doi.org/10.1080/10824669.2024.2342779>
4. Wilson, S. J., Lipsey, M., Tanner-Smith, E. E., Huang, C. H., & Steinka-Fry, K. T. (2011). Dropout prevention and intervention programs: effects on school completion and dropout among school-aged children and youth: a systematic review. *Campbell Systematic Reviews, 8*, 1–62. <https://doi.org/10.4073/csr.2011.8>
5. Wozney, L. M. (2009). *A systematic review of instructional interventions to improve school completion: mapping the evidence.* [Doctoral dissertation, Concordia University]. Concordia University Library. <https://concordiauniversity.on.worldcat.org/search?queryString=ot:(Spectrum)+976262>
6. Zaff, J. F., Donlan, A., Gunning, A., Anderson, S. E., McDermott, E., & Sedaca, M. (2017). Factors That Promote High School Graduation: A Review of the Literature. *Educational Psychology Review*. *29*(3): 447-76. <https://doi.org/10.1007/s10648-016-9363-5>

**List of seed articles**

Hartmann, T., Good, D., & Edmunds, K. (2011). Exito: Keeping High-Risk Youth on Track to Graduation through Out-of-School Time Supports. *Afterschool Matters*, *14*, 20-29.

Hemelt, S. W., Lenard, M. A., & Paeplow, C. G. (2019). Building bridges to life after high school: Contemporary career academies and student outcomes. *Economics of Education Review*, *68*, 161-178.

Heppen, J. B., Zeiser, K., Holtzman, D. J., O'Cummings, M., Christenson, S., & Pohl, A. (2018). Efficacy of the Check & Connect mentoring program for at-risk general education high school students. *Journal of Research on Educational Effectiveness*, *11*(1), 56-82.

Mac Iver, M. A., Stein, M. L., Davis, M. H., Balfanz, R. W., & Fox, J. H. (2019). An efficacy study of a ninth-grade early warning indicator intervention. *Journal of Research on Educational Effectiveness*, *12*(3), 363-390.

Marshall, J. H., Aguilar, C. R., Alas, M., Castellanos, R. R., Castro, L., Enamorado, R., & Fonseca, E. (2014). Alternative education programmes and middle school dropout in Honduras. *International Review of Education*, *60*, 51-77.

Sepanik, S., Zhu, P., Shih, M. B., & Commins, N. (2021). First-Year Effects of Early Indicator and Intervention Systems in Oregon. REL 2021-097. *Regional Educational Laboratory Northwest*.

Sletten, M. A., Tøge, A. G., & Malmberg-Heimonen, I. (2023). Effects of an early warning system on student absence and completion in Norwegian upper secondary schools: a cluster-randomised study. *Scandinavian Journal of Educational Research*, *67*(7), 1151-1165.

Somers, C. L., Wang, D., & Piliawsky, M. (2016). Effectiveness of a combined tutoring and mentoring intervention with ninth-grade, urban black adolescents. *Journal of Applied School Psychology*, *32*(3), 199-213.

Stohlman, S., Huang, F., & Cornell, D. (2022). High school graduation outcomes of student threat assessment. *Preventing School Failure: Alternative Education for Children and Youth*, *66*(2), 109-117.

Weinstein, J., Villares, E., & Brigman, G. (2021). The effect of the Student Success Skills small group intervention on factors associated with dropout potential. *The Journal for Specialists in Group Work*, *46*(3), 256-271.

**Annex 5: Draft codebook based on PICOS framework**

| **Characteristics** | **Levels and definitions** |
| --- | --- |
| **Population** | |
| Country | Where the study was conducted. Type “Not reported” if the study does not provide the information. Countries will be then grouped into the following geographical areas: Africa, Asia, Australia, Europe, North America, South America. |
| Grade level | School level at which the study was conducted (multiple options are allowed).  ECEC = Early childhood education and care (i.e., pre-K and K)  Elementary school = Grades 1-5  Middle school = Grades 6-8  Secondary school = Grades 9-12  Other (describe) = For compensation programs type the students’ age range. |
| Socio-economic status | The percentage of students receiving free and reduced-price lunch. If another measure is used, (e.g., income) type a brief description. Type “Missing” if the study does not provide the information. The following categories will be then created based on percentages: low SES, average/high SES. |
| Target population | The target population of the program (multiple options are allowed).  Students  Teen parents  Teachers  Parents  School leaders  Other (describe) |
| Urbanicity | Communities in which the study was conducted (multiple options are allowed). Type “Missing” if the study does not provide the information.  Urban  Suburban  Rural  Not reported |
| **Intervention** | |
| Program domain | Prevention  Intervention  Compensation |
| Program type | Categories described in Annex 1 |
| **Comparison** | |
| Counterfactual | Type of program implemented in the control group during the study (multiple options are allowed).  Business as usual  Alternative program  Other (describe) |
| **Outcome** | |
| Outcome domain | The outcome domain measured in the study (multiple options are allowed).  Ultimate outcomes  Intermediate outcomes |
| Outcome type (for intermediate outcomes) | The outcome type measured in the study (multiple options are allowed).  Categories described in Annex 2 |
| **Study design** | |
| Study design | MA = Meta-analysis  RCT = Randomized Controlled Trial  Matched = Matched control group with matching conducted prior to or after the program implementation  DiD = Difference-in-Difference  RDD = Regression Discontinuity Design  IV = Instrumental Variable |
| Baseline equivalence for QEDs | Demographic equivalence = Equivalence based on demographic characteristics (e.g., SES, ethnicity, risk factors)  No demographic equivalence = No baseline equivalence reported or established (i.e., demographics greater than 0.25 SD)  Pretest equivalence = Equivalence at pretest of the outcomes evaluated in the study  No pretest equivalence = No baseline equivalence reported or established for pretest (i.e., pretest difference greater than 0.25 SD) |
| Publication status | Published = papers, books, chapters  Unpublished = reports, white papers, conference papers, posters, dissertations |
| Study size for meta-analyses | Number of studies included for meta-analyses |
| Study size for all studies | For RCTs and QEDs, number of students in treatment and control conditions (possibly analytical sample).  For meta-analyses, overall number of students involved in the included studies, if reported; otherwise, type “Missing” |

**References of the Annexes**

Chappell, S. L., O'Connor, P., Withington, C., & Stegelin, D. A. (2015). *A Meta-Analysis of Dropout Prevention Outcomes and Strategies. A Technical Report in Collaboration with the Center of Educational Partnerships at Old Dominion University.* Clemson, SC: National Dropout Prevention Center/Network at Clemson University. <https://dropoutprevention.org/wp-content/uploads/2022/11/A-Meta-Analysis-of-DOP-Outcomes-and-Strategies-Chappell-et-al-2015.pdf>

Council Recommendation of 28 November 2022 on Pathways to School Success and replacing the Council Recommendation of 28 June 2011 on policies to reduce early school leaving (Text with EEA relevance) 2022/C 469/01. (2022). *Official Journal*, C 469, 1-15. CELEX: <https://eur-lex.europa.eu/legal-content/EN/TXT/?uri=CELEX:32022H1209(01)> [legislation]

European Education and Culture Executive Agency, Eurydice (2014). *Tackling early leaving from education and training in Europe: strategies, policies and measures*. Publications Office. <https://data.europa.eu/doi/10.2797/33979>

Johansson, B. (2019). *Dropping Out of School - A Systematic and Integrative Research Review on Risk Factors and Interventions*. Örebro University. <http://urn.kb.se/resolve?urn=urn:nbn:se:oru:diva-77853>

Kis, V. (2020). Improving evidence on VET: Comparative data and indicators. *OECD Social, Employment and Migration Working Papers n. 250*. Paris: OECD Publishing. <https://doi.org/10.1787/d43dbf09-en>.

Lippman, L. H., Ryberg, R., Carney, R., & Moore, K. A. (2015). Workforce Connections: Key “soft skills” that foster youth workforce success: toward a consensus across fields. *Child Trends Publication*, *56*. <http://hdl.voced.edu.au/10707/367556>

National Dropout Prevention Center/Network (2022). *Effective Strategies.* National Dropout Prevention Center/Network. <https://dropoutprevention.org/effective-strategies/>

Rumberger, R. W. (2011). Dropping out: Why students drop out of high school and what can be done about it. Harvard University Press. <https://doi.org/10.4159/harvard.9780674063167>

Rumberger, R., Addis, H., Allensworth, E., Balfanz, R., Bruch, J., Dillon, E., Duardo, D., Dynarski, M., Furgeson, J., Jayanthi, M., Newman-Gonchar, R., Place, K., & Tuttle, C. (2017). Preventing drop-out in secondary schools (NCEE 2017-4028). Washington, DC: National Center for Education Evaluation and Regional Assistance (NCEE), Institute of Education Sciences, U.S. Department of Education. <https://whatworks.ed.gov>

Shea, B. J., Reeves, B. C., Wells, G., Thuku, M., Hamel, C., Moran, J., Moher, D., Tugwell, P., Welch, V., Kristjansson, E., & Henry, D. A. (2017). AMSTAR 2: a critical appraisal tool for systematic reviews that include randomised or non-randomised studies of healthcare interventions, or both. *BMJ, 2017*(358), j4008. <http://doi.org/10.1136/bmj.j4008>

Wilson, S. J., Tanner‐Smith, E. E., Lipsey, M. W., Steinka‐Fry, K., & Morrison, J. (2011). Dropout prevention and intervention programs: Effects on school completion and dropout among school‐aged children and youth. *Campbell Systematic Reviews, 7*(1), 1-61. <https://doi.org/10.4073/csr.2011.8>
